# The Non-Fimbriate Phenotype Is Predominant among *Salmonella enterica* Serovar Choleraesuis from Swine and Those Non-Fimbriate Strains Possess Distinct Amino Acid Variations in FimH

**DOI:** 10.1371/journal.pone.0151126

**Published:** 2016-03-14

**Authors:** Chien-An Lee, Kuang-Sheng Yeh

**Affiliations:** 1 Department of Veterinary Medicine, School of Veterinary Medicine, College of Bio-Resources and Agriculture, National Taiwan University, Taipei, Taiwan; 2 National Taiwan University Veterinary Hospital, Taipei, Taiwan; Animal and Plant Health Agency, UNITED KINGDOM

## Abstract

Although most *Salmonella* serovars are able to infect a range of animal hosts, some have acquired the ability to cause systemic infections of specific hosts. For example, *Salmonella enterica* serovar Choleraesuis is primarily associated with systemic infection in swine. Adherence to host epithelial cells is considered a prerequisite for initial infection, and fimbrial appendages on the outer membrane of the bacteria are implicated in this process. Although type 1 fimbriae encoded by the *fim* gene cluster are commonly found in *Salmonella* serovars, it is not known whether *S*. Choleraesuis produces this fimbrial type and if and how fimbriae are involved in pathogenesis. In the present study, we demonstrated that only four out of 120 *S*. Choleraesuis isolates from pigs with salmonellosis produced type 1 fimbriae as assayed by the yeast agglutination test and electron microscopy. One of the 116 non-type 1 fimbria-producing isolates was transformed with plasmids carrying different *fim* genes from *S*. Typhimurium LB5010, a type 1 fimbria-producing strain. Our results indicate that non-type 1 fimbria-producing *S*. Choleraesuis required only an intact *fimH* to regain the ability to produce fimbrial appendages. Sequence comparison revealed six amino acid variations between the FimH of the non-type 1 fimbria-producing *S*. Choleraesuis isolates and those of the type 1 fimbria-producing *S*. Choleraesuis isolates. *S*. Choleraesuis that produced type 1 fimbriae contained FimH with an amino acid sequence identical to that of *S*. Typhimurium LB5010. Site-directed mutagenesis leading to the replacement of the non-conserved residues revealed that a change from glycine to valine at position of 63 (G63V) resulted in a non-type 1 fimbria-producing *S*. Choleraesuis being able to express type 1 fimbriae on its outer membrane. It is possible that this particular amino acid change prevents this polypeptide from proper interaction with other Fim subunits required for assembly of an intact type 1 fimbrial shaft in *S*. Choleraesuis; however, it remains to be determined if and how the absence of type 1 fimbriae production is related to the systemic infection of the swine host by *S*. Choleraesuis.

## Introduction

The *Salmonella* genus is composed of *Salmonella enterica* and *Salmonella bongori* species, which comprise six subspecies (I, II, IIa, IIb, IV, and V) subdivided into about 2800 serovars [1]. Most *Salmonella* serovars that are pathogenic to human and animals belong to the species *Salmonella enterica*. Many serovars infect a broad range of animal hosts and cause gastroenteritis, but some serovars are highly virulent in specific hosts [2]. For example, *Salmonella enterica* serovar Choleraesuis is primarily associated with systemic invasive infection in swine, but may sometimes infect other animals, including humans [3].

Fimbriae are hairlike appendages on the outer membrane of many *Enterobacteriaceae* and are involved in adherence to host epithelial cells, a prerequisite for infection. Although at least 13 gene clusters within *Salmonella* may have the potential to produce fimbrial appendages, type 1 fimbriae are the most commonly found type and are easily detected in vitro [4, 5]. Some fimbrial proteins can only be detected by flow cytometry from those samples that have been recovered 8 h after infection of bovine ligated ileal loops with *S*. Typhimurium [6]. The phenotypic expression of type 1 fimbriae is primarily a result of cooperation among several Fim proteins and arginine tRNA encoded by *fimU* within the *fim* gene cluster. FimA, FimI, FimH, and FimF are fimbrial subunits that form the shaft of type 1 fimbriae, with FimA as the major fimbrial subunit [7]. Type 1 fimbriae adhere to a variety of cells that possess mannose residues, such as erythrocytes, leukocytes, yeast, and respiratory cells [8]. This binding capacity is conferred by the FimH adhesin [9]. FimC and FimD are a chaperone and usher protein, respectively, whose respective functions are to assist the fimbrial subunit to the outer membrane of the cell and to assemble type 1 fimbriae in the proper order [10]. FimZ, FimY, STM0551, FimW, and *fimU* are involved in a delicate regulatory circuit for the production of type 1 fimbriae [11–15]. The precise contribution of type 1 fimbriae to virulence is still unclear because several factors, such as the *Salmonella* strain used, the inoculation route, and the laboratory animal chosen, affect the results [16, 17]. Nonetheless, this type of fimbria may play an important role at some stage of the infectious cycle of *Salmonella* [18].

Recent genome sequencing studies of host-adapted, invasive *Salmonella* serovars, including Choleraesuis, have revealed that extensive deletions and truncations occur in these serovars [19, 20]. The majority of the lost genes have functional counterparts in systemically noninvasive serovars; for example, pseudogenes have been found in most fimbrial clusters, including *fim* in serovar Typhi [21]. Since information regarding the type 1 fimbriae of *S*. Choleraesuis is limited, the present study investigated the distribution of the expression of fimbrial appendages among *S*. Choleraesuis isolates from diseased swine in the field. We report that most *S*. Choleraesuis isolates did not produce type 1 fimbriae and that allelic variation in *fimH* could be one of the reasons for this non-fimbriate phenotype.

## Materials and Methods

### Bacterial strains and plasmids

The *S*. Choleraesuis strains were originally isolated from pigs with a tentative diagnosis of swine salmonellosis in swine farms at different locations of Taiwan. The pigs with clinical signs had either died recently or had been euthanized by electric shock and examined post-mortem by a registered veterinarian. Specimens from liver, spleen, or lung were obtained by the veterinarian and brought back to the laboratory and cultured for *Salmonella* on Xylose Lysine Desoxycholate Agar (Difco/Becton Dickinson, Franklin Lakes, NJ). Suspected *Salmonella* colonies were initially screened by *Salmonella* O antiserum poly A-I, and Vi (Difco/Becton Dickinson). *S*. Choleraesuis were identified by *Salmonella* O Group C1 antiserum (Difco/Becton Dickinson) and then confirmed by polymerase chain reaction (PCR) analysis according to the protocol reported by Chiu et al. [22], and stored at -80°C. The major sources of our *Salmonella* were collected from swine farms located in Taichung, Tainan, Chiayi, Pingtung, and Changhua counties in Taiwan from 2001 to 2015. Each isolate stocked was from one swine. One hundred and twenty strains were selected from our stock (n = 185) by simple random sampling and included in this study. The other specified bacterial strains and plasmids used are listed in Table 1.

**Table 1:** Bacterial strains and plasmids used in this study.

Strain or plasmid	Genotype or relevant features	Reference or source
*Salmonella* Typhimurium		
LB5010	a LT2 strain derivative, Fim^+^ and fimbrial phase variable	[23]
*Salmonella* Choleraesuis		
SC-15, 27, 30, 46, 77	Clinical isolate, Fim^-^	This study
SC-31, 34, 39	Clinical isolate, Fim^+^ and fimbrial phase variable	This study
*Escherichia coli*		
DH5α		[24]
Plasmids		
yT&A	2.7 kb cloning vector that contains T7 promoter; Am^r^	Yeastern, Taiwan
pACYC184	4.2 kb cloning vector; Tet^r^ and Cm^r^	ATCC
pISF101	12.8 kb DNA with the complete *fim* genes of *S*. Typhimurium cloned into pACYC184	[25]
pfimAICDHF	8.0 kb *fimAICDHF* DNA fragment cloned into pACYC184; Cm^r^	This study
pfimZY0551WU	3.7 kb *fimZY0551WU* DNA fragment cloned into pACYC184; Cm^r^	This study
pfimAI	1.4 kb *fimAI* DNA fragment cloned into pACYC184; Cm^r^	This study
pfimAICD	6.1 kb *fimAICD* fragment cloned into pACYC184; Cm^r^	This study
pfimHF	1.6 kb *fimHF* fragment cloned into pACYC184; Cm^r^	This study
pfimH	1.2 kb *fimH* DNA fragment cloned into pACYC184; Cm^r^	This study
pfimHL57P	A *fimH* coding sequence with L57P cloned into pACYC184; Cm^r^	This study
pfimHG63V	A *fimH* coding sequence with G63V cloned into pACYC184; Cm^r^	This study
pfimHR89Q	A *fimH* coding sequence with R89Q cloned into pACYC184; Cm^r^	This study
pfimHA115T	A *fimH* coding sequence with A115T cloned into pACYC184; Cm^r^	This study
pfimHS131Y	A *fimH* coding sequence with S131Y cloned into pACYC184; Cm^r^	This study
pfimHG177S	A *fimH* coding sequence with G177S cloned into pACYC184; Cm^r^	This study

### Yeast agglutination test

The expression of type 1 fimbriae on the bacterial surface was analyzed by the yeast agglutination test [26]. Briefly, the bacterial isolate was cultured in 10-mL of Luria-Bertani (LB) broth (Difco/Becton Dickinson, Franklin Lakes, NJ) at 37°C for 48 h statically or grown on LB agar (Difco/ Becton Dickinson) at 37°C for 24 h. Cells were pelleted from broth by centrifugation for 10 min or scraped from agar and resuspended in 1 × phosphate-buffered saline. Aliquots of bacterial suspension and 3% *Saccharomyces cerevisiae* (Sigma-Aldrich, St. Louis, MO) were mixed on a glass slide and gently agitated for 1 min. Visible agglutination indicated the presence of type 1 fimbriae. Positive samples were mixed again with yeast cells and 3% D-mannose solution (Sigma-Aldrich). Mannose-sensitive agglutination conferred by type 1 fimbriae was indicated by inhibition of agglutination in the presence of mannose.

### DNA manipulation

#### Complementation test

The primers used for amplification are listed in Table 2. The template was genomic DNA extracted from SC-39, a Fim^+^ strain with a commercial kit (GeneMark, Taipei, Taiwan). Briefly, the PCR mixture contained 1 μg of template DNA, 10 mM of primers, 2.5 mM of dNTP, 5 × Phusion High Fidelity Buffer, and 1 unit of Phusion DNA polymerase (ThermoFisher Scientific, Wltham, Massachusetts, USA). The PCR conditions consisted of initial denaturation at 98°C for 3 min, followed by 35 cycles at 98°C for 30 s, 50°C for 30 s, and 72°C for 30 s/kb, and extended at 72°C for 10 min. The PCR products were cleaned with a GeneJet PCR purification kit (ThermoFisfer Scientific). Amplified DNA fragments were incubated at 37°C for 30 min with 1 unit of *Taq* DNA polymerase to add an adenine residue before the subsequent TA-cloning step. Insert DNA was cloned into the T&A Cloning Vector by blue-white selection according to the protocol provided by the manufacturer (Yeastern Biotech, Taipei, Taiwan). The recombinant plasmid was isolated with a Plasmid Miniprep Purification kit (GeneMark), and the insert DNA was sequenced (Mission Biotech, Taipei, Taiwan) to confirm that no error had been introduced during PCR. The insert in the plasmid was retrieved after cleavage with *Bam*HI and *Sph*I (FastDigest, ThermoFisher Scientific) and cloned into vector pACYC184. The recombinant plasmid was introduced into the Fim^-^ SC-15 strain by electroporation (ECM 830 Electroporation System, Harvard Apparatus, Holliston, Massachusetts, USA). The transformants were tested for their ability to produce type 1 fimbriae with the yeast agglutination test.

**Table 2:** Primers used in this study.

primer	Sequence 5’-3’
fimAICDHF-F	ACGGATGAGGATCCACGTTTGCTTGCGACATAAATCTGTGA
fimAICDHF-R	TTCAGCGTGCATGCTCTGGCCAATGAAATGTCTAACAAAGA
fimZY0551WU-F	ATTGACTGGGATCCTGATCAATTACAATTAGTGTCCGTTATT
fimZY0551WU-R	CTCAAGAGGCATGCCGAAAATAAAAATAGAAGACTTTCGCTT
fimAI-R	CTCAAGAGGCATGCGGCGTCTGCGGCAAATT
fimAICD-R	CTCAAGAGGCATGCCAAGCCGCATCGATAAAT
fimHF-F	AGATGAGGATCCATGAAAATATACTCAGCGCTATTG
fimHF-R	TAGCGTGCATGCCTAATTGTAATTGATCAGGAAGTTC
fimH-R	TAGCGTGTCGACAATATTCACTTCGCCCAGAGATGAG
L57P-F	GTGGTTACGCTGCCGGAAAAATCAGGTTGG
L57P-R	CCAACCTGATTTTTCCGGCAGCGTAACCAC
G63V-F	AAATCAGGTTGGGTCGGCGTAAACGCG
G63V-R	CGCGTTTACGCCGACCCAACCTGATTT
R89Q-F	GAATTACGGGTACAAAGCACCGAAGGAAAT
R89Q-R	ATTTCCTTCGGTGCTTTGTACCGGTAATTC
A115T-F	ACCGATAGTGTCACTGGGGTATTTTAT
A115T-R	ATAAAATACGCCAGTCACACTATCGGT
S131Y-F	ATGGGCGTCGACTATAACGTGTCGCAGCAA
S131Y-R	TTGCTGCGACACGTTATAGTCGACGCCCAT
G177S-F	ACGACCTCTACCAGCGACGCGTTGAGCACG
G177S-R	CGTGCTCAACGCGTCGCTGGTAGAGGTCGT

#### Site-directed mutagenesis of *fimH*

A mutant allele of *fimH* was generated by site-directed mutagenesis using an overlapping-extension PCR with SC-15 strain genomic DNA template and mutagenic oligonucleotide primers L57P-F and L57P-R [27]. Briefly, fimH-F and L57P-R were used to amplify the first DNA fragment using Phusion DNA polymerase. The PCR conditions consisted of initial denaturation at 98°C for 30 s, followed by 35 cycles at 98°C for 10 s, 50°C for 30 s, and 72°C for 40 s. The second DNA fragment was amplified using fimH-R and L57P-F primers by the same PCR conditions described above. These two DNA fragments were purified with a GeneJet PCR Extraction Kit. Ligation of these two DNA fragments with two overlapping ends was achieved with fimH-F and fimH-R primers as follows: denaturation at 98°C for 30 s, ligation at 50°C for 30 s, and elongation at 72°C for 45 s, followed by 35 cycles at 98°C for 10 s, 50°C for 30 s, and 72°C for 45 s. The amplified fragments were cloned into the T&A Cloning Vector and sequenced to determine whether the codon encoding leucin at amino acid 57 had been replaced with proline. The other *fimH* alleles were constructed with the same methods. The insert DNA was cloned into the pACYC184 vector and transformed into a SC-15 strain by electroporation and tested for its ability to produce type 1 fimbriae as described above.

### Electron microscopy

The bacterial strains were resuspended in ddH_2_O, and 20 μL was taken to mix with 20 μL of 2% phosphotungstic acid in a microcentrifuge tube. A drop of this mixture was placed onto a formvar grid and left for 20 s. Excess fluid was removed with filter paper. The grid was observed with a JEOL JEM-1400 transmission electron microscope (JEOL Ltd, Tokyo, Japan).

## Results

Among the 120 *S*. Choleraesuis isolates screened for type 1 fimbrial expression with the yeast agglutination test, only four produced type 1 fimbriae (4/120 = 3.3%). Because many *Salmonella* isolates exhibited multidrug-resistant phenotypes, which would interfere with our further cloning studies, all of these Fim^-^ isolates were tested for chloramphenicol sensitivity using the Kirby-Bauer method and interpreted according to the CLSI guidelines [28]. We found five *S*. Choleraesuis isolates that were chloramphenicol sensitive and thus potential recipients of recombinant pACYC184 plasmids. One of these isolates, SC-15, was chosen for complementation tests.

**Fig 1:**
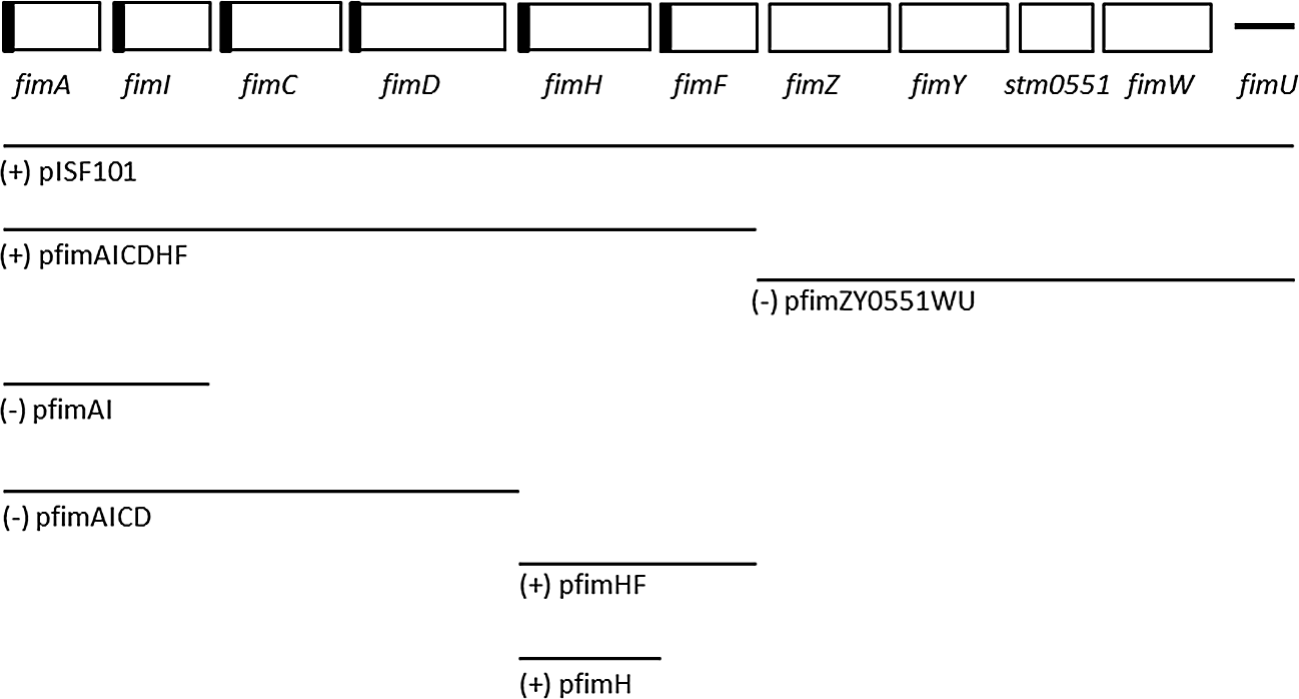
Summary of the yeast agglutination test results of SC-15 transformed with different recombinant plasmids. The signal peptide regions of the structural and biosynthetic *fim* genes are shown as filled black boxes. Solid lines indicate the *fim* gene(s) retained on the pACYC184. (+): positive result in the yeast agglutination test, (-): negative result in the yeast agglutination test.

To determine whether the SC-15 type 1 fimbria-negative strain carried defects in the *fim* gene cluster, pISF101 and different derivatives from the *fim* of SC-39 were introduced into SC-15. Transformants that carried pACYC184 only did not produce type 1 fimbriae. pISF101, which possessed the entire *fim* gene cluster, allowed SC-15 to produce type 1 fimbriae and mediate yeast agglutination. To further dissect which structural or regulatory portion of the *fim* gene cluster was responsible for fimbrial expression in SC-15, pfimAICDHF and pfimZY0551WU were constructed and it was revealed that the SC-15 transformed with pfimAICDHF had fimbriae. On the basis of this finding, pfimAI, pfimAICD, pfimHF, and pfimH were further constructed to test which elements of the structural genes would permit SC-15 to produce type 1 fimbriae. We found that plasmids that carried fimHF or fimH alone were able to produce a fimbriate phenotype in SC-15 (Fig 1).

Since FimH confers binding specificity for type 1 fimbriae [29], *fimH* of non-fimbriate isolates SC-15, 27, 30, 46, and 77 and that of fimbriate isolates SC-31, 34, and 39 were sequenced. The FimH of *S*. Typhimurium LB5010 (Fim^+^) and *S*. Choleraesuis SC-B67 (Fim^-^) [19] were also aligned for comparison. It was interesting to find that the Fim^-^ *S*. Choleraesuis isolates and SC-B67 strain possessed the same FimH sequences, whereas those of FimH from Fim^+^ *S*. Choleraesuis isolates and *S*. Typhimurium LB5010 were identical. The difference between the Fim^+^ and Fim^-^ groups resides in only six amino acid residues: Fim^+^/Fim^-^ (position): P/L (57), V/G (63), Q/R (89), T/A (115), Y/S (131), and S/G (177) (Table 3).

**Table 3:** Amino acid variations in FimH of Fim^+^ and Fim^-^ strains.

Strain\ position	57	63	89	115	131	177
LB5010 (Fim^+^)	P	V	Q	T	Y	S
SC-31(Fim^+^)	P	V	Q	T	Y	S
SC-34 (Fim^+^)	P	V	Q	T	Y	S
SC-39 (Fim^+^)	P	V	Q	T	Y	S
SC-B67 (Fim^-^)	L	G	R	A	S	G
SC-15 (Fim^-^)	L	G	R	A	S	G
SC-27 (Fim^-^)	L	G	R	A	S	G
SC-30 (Fim^-^)	L	G	R	A	S	G
SC-46 (Fim^-^)	L	G	R	A	S	G
SC-77 (Fim^-^)	L	G	R	A	S	G

**Fig 2:**
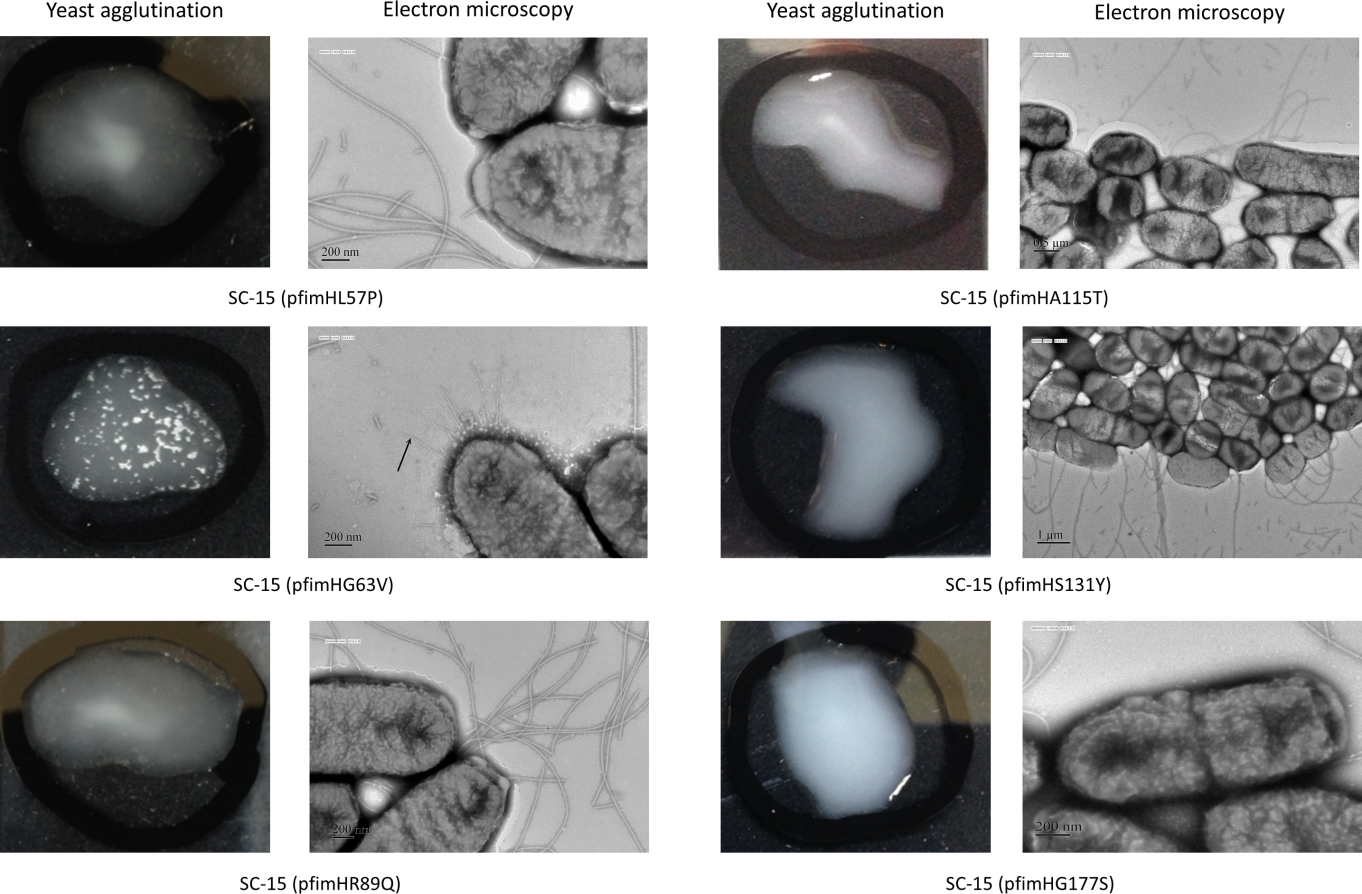
Results of the yeast agglutination test and electron microscopy. SC-15 transformed with pfimHG63V enabled the mediation of yeast agglutination and produced fimbrial appendages (arrow) on the outer membrane of the bacterial cell. Other transformants did not mediate yeast cells to agglutinate, and no fimbrial structures other than flagella were observed.

The introduction of plasmids with *fimH* inserts carrying the various amino acid changes demonstrated that only the SC-15 transformant that carried pfimHG63V changed the phenotype to Fim^+^ (Fig 2).

## Discussion

*S*. Choleraesuis is the most common cause of swine salmonellosis and infection of these animals manifests primarily as septicemia, whereas *S*. Typhimurium accounts for most cases of enterocolitis in swine [30, 31]. The type 1 fimbriae of *S*. Typhimurium bind to enterocytes of swine in vivo [32], but little information regarding this type of fimbriae in *S*. Choleraesuis has been reported. In this study, we found that most *S*. Choleraesuis isolates from diseased swine (116 of 120) did not mediate yeast agglutination. Appendages like type 1 fimbriae in nontyphoidal *Salmonella* serovars may play an important role in inducing the inflammatory response during the course of intestinal infection, thus facilitating the survival of fimbriated *Salmonella* in such a milieu and restricting infection to the gastrointestinal tract [33]. In contrast, some host-restricted *Salmonella* serovar, such as Gallinarum, are deficient in mannose-sensitive binding activity [34–36], instead, they produce morphologically similar fimbriae called type 2 fimbriae, with a preferable binding ability to avian leukocyte than to mammalian cells [37]. These differences in binding specificity are primarily associated with allelic variations in FimH adhesins [36, 38]. *S*. Choleraesuis that are deprived of type 1 fimbriae are perhaps unable to colonize the intestinal mucosa. They might cause less of an inflammatory response and are able to enter the bloodstream more rapidly than strains expressing type 1 fimbriae. Most *Salmonella* serovars isolated from swine carcasses, live swine, or their environments are primarily those with a broad host range, such as Typhimurium, Derby, and Anatum, whereas Choleraesuis has only rarely been identified as predominant serovar [39, 40].

Although proteins other than Fim proteins, such as leucine-responsive regulatory protein, were also found to modulate type 1 fimbrial expression [41], we focused only on the *fim* gene cluster. Our results demonstrated that *fimH* from *S*. Typhimurium was uniquely able to cause a Fim+ phenotype in strain SC-15. In contrast, *E*. *coli* which has been reported to have the ability to assemble a fimbrial shaft even in the absence of FimH [42], *S*. Choleraesuis was unable to produce type 1 fimbriae without FimH. This finding is in line with the report of Zeiner et al. that indicated that FimA, FimF, and FimH are necessary for the assembly of type 1 fimbriae in *Salmonella* [7].

It has been postulated that the repeated occurrence of phylogenetically unlinked mutations at the same amino acid positions, or hot spot mutations, may represent evidence of adaptive evolution via the molecular convergence of protein variants [43]. A comparison of the amino acid sequences of FimH alleles from Fim^+^ and Fim^-^ *S*. Choleraesuis isolates revealed that all six amino acid differences between these two groups were included in the previously described hot spot mutations in the *fimH* of a large set of *Salmonella* strains [44, 45]. Fim^+^/Fim^-^ (position): P/L (57), V/G (63), and Q/R (89) were identified as recent structural mutations, indicating that they have emerged only recently, whereas Y/S (131) is a long-term structural mutation that occurred in protein variants that are persistent in nature over long evolutionary periods [45, 46]. Positions 115 and 177 of Fim^-^ *S*. Choleraesuis and SC-B67 strain were A and G, respectively; whereas those positions were T and S in Fim^+^ *S*. Choleraesuis and *S*. Typhimurium LB5010. Interestingly, we found that only FimH of *S*. Schwarzengrund exhibited T and S; all others showed A and G at these positions according to the current Microbial Variome Database provided by Dr. Sokurenko (http://depts.washington.edu/sokurel/variome/). Nevertheless, a switch between these two amino acids in different *fimH* alleles at position 115 or 177 did not have an impact in terms of fimbrial production in our study.

Site-directed mutagenesis analysis showed that only one amino acid substitution in FimH (G63V) was sufficient to restore the ability of SC-15 to produce type 1 fimbriae. This finding correlated with observations in *S*. Gallinarum. The FimH of *S*. Gallinarum and *S*. Typhimurium only differ by six residues, and one amino acid substitution (I78T) in the hot spot mutation zone was sufficient to restore the ability of serovar Gallinarum to gain mannose-specific binding activity [35, 47]. The ability of *Salmonella* to colonize or cause diseases in different hosts probably depends not only on the presence of an array of specific genes, but also on the allelic variation within these genes [38]. In our study, the lack of capability to produce type 1 fimbriae is not likely a result of the absence of FimH, but it nonetheless may be attributable to the property of the FimH polypeptides [48]. The amino acid variations in SC-15 are located at the N-terminal lectin-domain of FimH. We can only hypothesize that glycine at residue 63 may interfere with the binding of the chaperone-adhesin FimC/FimH complex to the N-terminal domain of usher protein FimD, which is the first step of type 1 fimbrial biogenesis in the well-studied *E*. *coli* model [49].

One limitation of our study was that most of the clinical *S*. Choleraesuis isolates from diseased swine were multidrug-resistant and could not be used in the same manner as strain SC-15. The fact that many *S*. Choleraesuis isolates were Fim^-^ could suggest that this phenotype could contribute to the virulence of this serovar in swine.

## Conclusions

Although the results from the present study were obtained with only one *S*. Choleraesuis isolate, it appears likely that allelic variation in *fimH* is a cause of the widespread nonfimbriate phenotype of *S*. Choleraesuis shown in this study. This hypothesis is supported by observation that the FimH amino acid sequences of the Fim^+^ *S*. Typhimurium LB5010 and Fim^-^ *S*. Choleraesuis SC-B67 differ only by 6 amino acids. Sequencing of FimH from more Fim^-^ *S*. Choleraesuis is currently underway in our laboratory.
